# Comparative Response of Canine and Human Osteosarcoma Tumour Cell Lines to Molecularly Targeted Anticancer Agents at Clinically Relevant Exposures With Analysis of Genomic Biomarkers

**DOI:** 10.1111/vco.70074

**Published:** 2026-04-26

**Authors:** Daniel L. Gustafson, Kala A. Early, Maritza A. Morales, Klaudia A. Poplawski, Sunetra Das, Dawn L. Duval

**Affiliations:** ^1^ Flint Animal Cancer Center, Veterinary Teaching Hospital Colorado State University Fort Collins Colorado USA; ^2^ Cell and Molecular Biology Graduate Program Colorado State University Fort Collins Colorado USA; ^3^ University of Colorado Cancer Center University of Colorado, Anschutz Medical Campus Colorado Aurora USA

## Abstract

Osteosarcoma (OSA) is a primary bone tumour occurring in children but is also prevalent in large breed dogs. Canine OSA (cOSA) has long been viewed as analogous to human OSA (hOSA) with cOSA serving as a surrogate for development of therapeutic approaches to treat the rarer human form. Drug therapy of OSA has remained virtually unchanged over the last four decades and drug testing is challenging due to the genomic heterogeneity of OSA as well as the limited number of patients for clinical trials. Thus, an approach that includes a suitable clinical surrogate at the early stage of therapeutic development may be beneficial. Therefore, to address these challenges, a phenotypic drug screen of 12 targeted anticancer drugs was carried out using 13 cOSA and 6 hOSA cell lines and responses compared at clinically relevant exposures (CRE) estimated from human data. The results identified four drugs (alisertib, crizotinib, onvansertib and sorafenib) with significant responses at the CRE in cOSA and hOSA cell lines and demonstrated that drug responses were indistinguishable across species. Correlations of drug response with genomic biomarkers in the cOSA cell line panel identified Myc and Hedgehog signalling as potential predictors of crizotinib response and Myc, epithelial markers and anti‐apoptotic signalling for onvansertib response. The conclusions of these findings are that cOSA and hOSA cell lines show the same range of response to targeted agents and identify potential biomarker pathways for further investigation in OSA tumours for use in future comparative oncology studies including clinical trials in pet dogs.

## 1 Introduction

Osteosarcoma (OSA) is a primary tumour of the bone that occurs in both humans and dogs. Human OSA (hOSA) occurs primarily in children and canine OSA (cOSA) in large breed dogs. In both, the tumour predominately originates in the appendicular as opposed to the axial skeleton and treatment involves surgical resection, including amputation, followed by adjuvant chemotherapy consisting of platinum‐ and anthracycline‐based chemotherapy with hOSA treatment also including methotrexate [1, 2]. Treatment of OSA in both human and canine patients has remained virtually unchanged since the 1980s with pulmonary metastasis being the primary point of disease progression and subsequent patient mortality.

The development of targeted anticancer drugs has focused on the development of therapeutics that inhibit proteins with activating mutations or target overexpression [3]. The success of the BCR‐ABL targeted agent imatinib in chronic myelogenous leukaemia is a clear example of the success of this approach [4]. However, this approach has been limited by signalling redundancy and bypass mechanisms leading to successful application where pathway addiction can be clearly demonstrated and inhibition leads to robust responses. Identification of specific mutations, pathway activation, and other genomic‐based biomarkers has led to the classification of tumours not just by histology but also by genomic classifier. For example, ERBB2 expressing breast cancers are treated with ERBB2‐targeted therapies [5] and genomic alterations in ERBB1, ALK, or NTRK in lung cancer are important biomarkers for response to the specific targeted agents gefitinib [6], crizotinib [7, 8] and Larotrectinib [9, 10]. The identification of responders and non‐responders, as well as genomic biomarkers that can be used as classifiers within patient populations, is the critical component to this type of approach and leads to significant increases in response as well as success in treatment development [11].

The genomic landscape in OSA, both human and canine, is characterized by high rates of structural variation and relatively few single‐nucleotide variants with the primary molecular changes being alterations in TP53 and TP53‐related pathways [12, 13, 14, 15]. The molecular background of cOSA cell lines is also heterogenous and dominated by TP53 [16]. This molecular heterogeneity makes it difficult to take an activating mutational driver approach to drug screening in OSA. The use of phenotypic screens of drug response across a panel of OSA cell lines allows for an unbiased and data‐based approach to first identify agents with activity against at least a subset of cell lines. Potential drivers may then be identified in responsive cell lines leading to unmasking of novel genomic alterations and potential biomarkers. Therefore, our initial studies with cell line panels of canine and human OSA are an attempt to establish these drug‐response relationships with genomic biomarkers in a species agnostic manner as a first step towards identifying targeted agents with potential activity in OSA.

## 2 Materials and Methods

### 2.1 Reagents and Chemicals

Alisertib (Cat# S1133), alpelisib (Cat# S2814), cabozantinib (Cat# S1119), crizotinib (Cat# S1068), dasatinib (Cat# S1021), defactinib (Cat# S7654), onvansertib (Cat# S7255), palbociclib (Cat# S4482), sapitinib (Cat# S2192), sorafenib (Cat# S7397), vactosertib (Cat# S7530), venetoclax (Cat# S8048) and volasertib (Cat# HY‐12137) were all purchased as 10 mM stocks in DMSO or in solid form from Selleck Chemicals (Houston, TX) or Med Chem Express (Monmouth Junction, NJ). Dulbecco's Modified Eagle's Medium (DMEM) (Cat# 10‐017‐CV, Corning, Corning, NY), Minimum Essential Medium (MEM) (Cat# 10–010‐CV, Corning, Corning, NY), McCoy's 5A (Cat# 10‐050‐CV, Corning, Corning, NY), Fetal Bovine Serum (FBS) (Cat# PS‐FB3, Peak Serum, Wellington, CO or Cat# 76419–584, Avantor, Radnor Township, PA), penicillin–streptomycin+l‐glutamine (Cat# 30‐009‐CI, Corning, Corning, NY), sodium pyruvate (Cat# 25‐000‐CI, Corning, Corning, NY) and HEPES pH 7.3 (Cat# J16924‐AP, ThermoFisher Scientific, Waltham, MA) were all purchased from indicated suppliers. YOYO‐1 (Cat# Y3601) was purchased from ThermoFisher Scientific (Waltham, MA).

### 2.2 Cell Lines and Cell Cultures

Unlabeled and transduced (Incucyte NucLight Red, Cat# 4625, Essen Bioscience Inc., Ann Arbor, MI) canine OSA cell lines were obtained from the cell repository at the Flint Animal Cancer Center. Cell lines were validated by microsatellite analysis [17] prior to use. Human cell lines were obtained from the CU Cancer Center Cell Technologies Shared Resource as authenticated and mycoplasma free stocks and transduced with Incucyte NucLight Red. All cell lines were maintained at 37°C and 5% CO_2_ in DMEM, MEM, or McCoy's supplemented with 10% FBS, penicillin–streptomycin, 1 mM sodium pyruvate and 10 mM HEPES pH 7.3. Cultured cells were used for experiments within 20 passages. The molecular characteristics of the cOSA and hOSA cell lines used in the drug screens are shown in Tables S1 and S2.

### 2.3 Calculation of the Clinically Relevant Exposure for Targeted Agents

The clinically relevant exposure (CRE) value was defined as the average plasma concentration at steady‐state (*C*_p,ss_) as estimated using the equation [18]

$$C_{p,\mathrm{ss}}=\frac{\text{Dose}\cdot F}{{\mathrm{CL}}_{\mathrm{ss}}\cdot \tau }$$

where *F* is the bioavailability, CL_ss_ represents clearance at steady‐state and *τ* is the dosing interval. If CL_ss_ was not calculated in the cited publications it was estimated as follows:

$$\frac{{\mathrm{CL}}_{\mathrm{ss}}}{F}=\frac{\text{Dose}}{{\mathrm{AUC}}_{\mathrm{ss},\;0-\tau }}$$

A description of the data used and the calculation of the CRE for each of the drugs is included in the Supporting Information (Calculation of Clinically Relevant Exposure).

### 2.4 Monitoring Proliferation and Cell Death

Dosing experiments were performed using the Opentrons OT‐2 Robot (Opentrons Labworks Inc., Long Island City, NY). Cells were plated out in 96‐well tissue culture plates (Nunclon Delta Surface, Cat# 167008, ThermoFisher Scientific, Waltham, MA) at a density of 200–1000 cells/well and allowed to attach. Plated cells were subsequently dosed at a ratio of 1:1 with 2× graded concentrations of each drug and media containing 200 nM YOYO‐1 dye for a final plated 1× graded concentrations of each drug and 100 nM YOYO‐1 dye. Dosed plates were scanned for 4 days using the Incucyte SX5 Live‐Cell Analysis System (Essen Bioscience Inc.) Live cell numbers in each well were determined by red object count (RFP expression) for transduced cell lines or total object count (Cell‐by‐Cell (CBC) analysis) for unlabeled cell lines. Dead cell numbers (YoYo positive) in each well were determined by green object count. The green object count was subtracted from total object count for a live cell count in unlabeled cell lines. Cell growth was monitored both as fraction of vehicle treated growth as well as through calculation of growth rate inhibition (Growth Rate Response) [19]. The calculation of growth rate (GR) response is based on measured growth rate of control and treated cells and is calculated at a given drug concentration (c) and time (*t*) as compared to vehicle treated cells (*v*) using the following equation:

$$\mathrm{GR}\left(c,t\right)=2^{k\left(c,t\right)/k\left(v,t\right)}-1$$

Drug concentrations that result in a 50% decrease in the GR response (GR_50_) were calculated by linear regression of a linear portion of the curve (*r* > 0.8) spanning the GR_50_ value (GR = 0.5).

### 2.5 RNASeq, GSVA and Pa241thway Score Calculation

Paired end RNA sequencing reads (150 bp, 31.3 to 65.2 million clusters) were mapped against CanFam3.1 (Ensembl version 99) with STAR using genecount parameters (v 2.6.1a). Gene count data was normalized using Deseq2 (v1.26.0) median of ratios method and RUVseq (RUVg method). Normalized count data was log‐transformed and scaled for plotting and downstream analyses. Gene set variation analysis was conducted using GSVA R Package (v. 1.52.3) with the RUVseq and the Hallmark Gene Set Collection from the Human MsigDB Collections.

### 2.6 Myc Score Calculation

The Myc score was calculated independently using 18 Myc target genes and a rank‐based scoring system as previously described [20]. Briefly, cOSA cell lines were given an integer score based on *Z* scores calculated from RNASeq counts across a 58‐cell line canine panel and the combined scores for the genes *Z* scored for a comparative Myc score. Genes upregulated by Myc were ranked from low to high, whereas genes downregulated by Myc were ranked from high to low.

### 2.7 EPI, MES and EMT Transcription Score Calculation

An EPI and MES score were calculated based on gene expression correlations with the EMT markers E‐cadherin (CDH1), vimentin (VIM), N‐cadherin (CDH2), and fibronectin 1 (FN1) [21] as previously described. Thirty‐five genes that correlated with CDH1 expression were used for calculation of the EPI score, and 33 genes that correlated with VIM, CDH2, and FN1 were used for calculation of the MES score. An EMT Transcription score was calculated based on the expression of the SNAIL (SNAI1), TWIST2, and ZEB1 transcription factors, which play a significant role in transcriptional regulation of EMT [22].

### 2.8 Anti‐Apoptotic Score

An anti‐apoptotic score based on a three gene signature including BCL2, MCL1, and BCL2L2 [23] was calculated using a rank based estimation as described above for the Myc score calculation.

### 2.9 Statistical Evaluation

Correlation analysis for continuous variables was carried out by Pearson correlation and for ranked variables by Spearman correlation. Multiple unpaired *t*‐tests were used to determine differences in mean drug response between the cOSA and hOSA cell lines. Two‐way ANOVA analysis followed by Tukey's multiple comparisons test was used for comparison of drug response across cell lines. Correlation analysis, multiple unpaired *t*‐tests and ANOVA analysis was done using GraphPad Prism version 10.5.0 for MacOS (GraphPad Software, Boston, MA). Bliss analysis for determining interaction between crizotinib and vismodegib was done using the SynergyFinder R package [24] ran on R version 4.4.3.

## 3 Results

### 3.1 CRE for Cell‐Based Studies

In vitro exposure of cells to drug levels like those seen in patients was estimated and termed CRE. Figure S1 shows the plasma levels of crizotinib and sorafenib, respectively, as reported in human pharmacokinetic studies at steady state within a dosing interval along with the estimated CRE. The putative drug targets as well as the tested range and CRE estimated for each of the 12 drugs and the drug concentration used to simulate the CRE in vitro are shown in Table 1. The CRE values ranged from a low of 14 ng/mL (28.7 nM) for dasatinib and a high of 4307 ng/mL (9.27 μM) for sorafenib.

**TABLE 1:** Drugs used in screen and pharmacokinetic parameters.

Drug	Putative target	Tested range (ng/mL)	CRE (ng/mL)^a^	In vitro CRE^b^ (ng/mL)
Alisertib	Aurora A Kinase	50–1500	1150	1000
Alpelisib	PI3K	15–500	550	500
Cabozantinib	MET, VEGFR 1, 2 and 3, AXL, RET, ROS1, TYRO3, MER, KIT, TRKB, FLT‐3, TIE‐2	100–2500	765	1000
Crizotinib	ALK, c‐MET, RON	2.5–200	415	400
Dasatinib	BCR‐ABL, SRC, c‐KIT, EPHA2, PDGFR‐β	5–150	14	15
Defactinib	FAK	100–4000	362	500
Onvansertib	Polo‐Like Kinase 1	10–600	109	100
Palbociclib	CDK 4/6	5–300	91	100
Sapitinib	ErbB1, B2, B3	25–1000	342	500
Sorafenib	RAF1, BRAF, VEGFR 1, 2 and 3, PDGFR, KIT, FLT3, FGFR1, RET	25–5000	4307	4000
Vactosertib	TGFBR1	50–1500	833	1000
Venetoclax	BCL‐2	50–2000	1204	1500

^a^ Clinically relevant exposure (CRE) was estimated as described in the Supporting Information.

^b^ In vitro CRE is the concentration used in cell culture studies to represent the estimated CRE.

### 3.2 Response in cOSA Cell Lines

**FIGURE 1:**
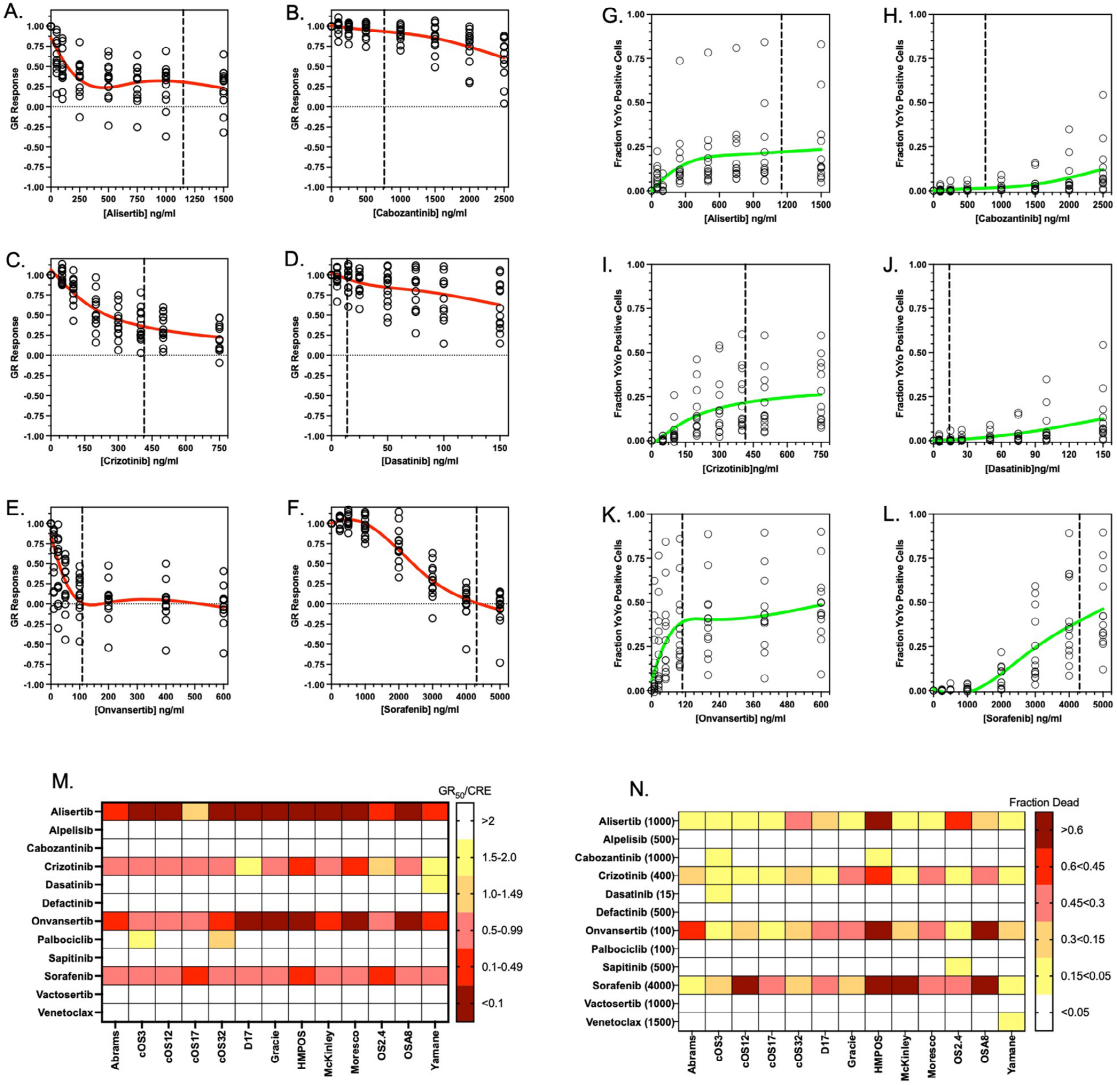
Growth rate (GR) response of cOSA cell lines to (A) alisertib, (B) cabozantinib, (C) crizotinib, (D) dasatinib, (E) onvansertib and (F) sorafenib following 96‐h continuous exposure to each drug. Open circles at each timepoint represent the average response of a given cell line and the red line represents the average spline curve for all cell lines combined. The vertical dashed line is drawn at the CRE value for each individual agent. Cytotoxic response as represented by the fraction of YoYo positive cells in cOSA cell lines to (G) alisertib, (H) cabozantinib, (I) crizotinib, (J) dasatinib, (K) onvansertib and (L) sorafenib sorafenib following 96‐h continuous exposure to each drug. Open circles at each timepoint represent the average response of a given cell line and the green line represents the average spline curve for all cell lines combined. The vertical dashed line is drawn at the CRE value for each individual agent. (M) Heat map representing the relationship of the measured GR_50_ and the CRE for 12 targeted agents and 13 cOSA cell lines. A lower GR_50_/CRE value represents greater sensitivity to a given agent relative to the CRE. (N) Heat map representing the fraction of dead cells at in vitro drug levels representing the CRE.

The GR response and cytotoxicity of targeted agents in 13 cOSA cell lines are shown in Figure 1. For comparison of response across cell lines and the drugs tested, the calculated GR_50_ value for each drug and cell line was divided by the CRE for each drug and displayed as a heatmap (Figure 1M). A value of 1 indicates that a 50% reduction in growth rate is observed at the CRE and values > 1 suggest little activity and values < 1 suggest significant activity. The analysis of this data shows that alisertib, crizotinib, onvansertib, and sorafenib are the only drugs within the tested set that show significant activity in most of the cell lines tested. This is also reflected in the measured cytotoxicity at drug concentrations reflective of the CRE shown in Figure 1N where significant fractions of dead cells are also observed with this same set of drugs.

### 3.3 Response in hOSA Cell Lines

**FIGURE 2:**
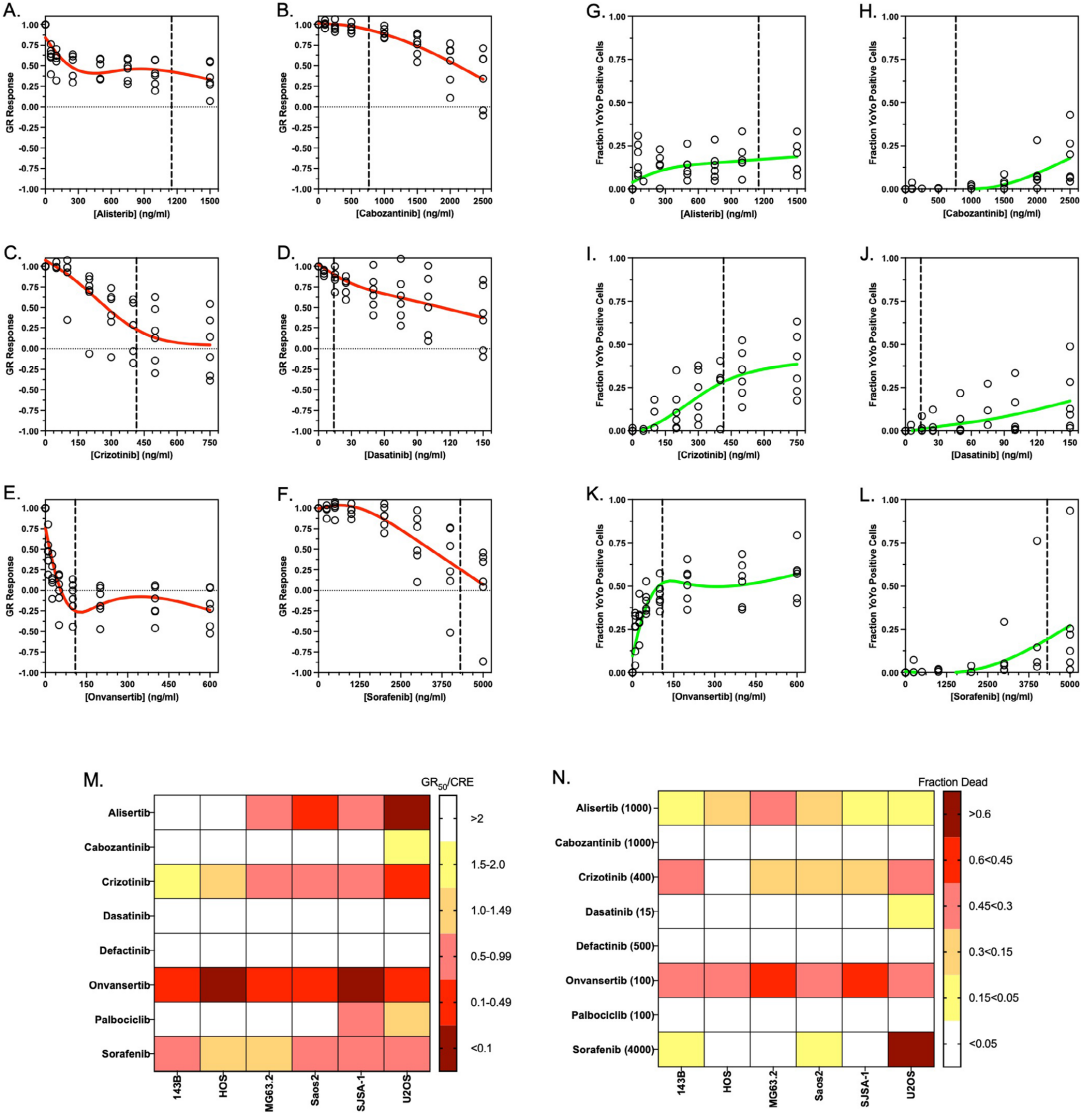
Growth rate (GR) response of hOSA cell lines to (A) alisertib, (B) cabozantinib, (C) crizotinib, (D) dasatinib, (E) onvansertib and (F) sorafenib following 96‐h continuous exposure to each drug. Open circles at each timepoint represent the average response of a given cell line and the red line represents the average spline curve for all cell lines combined. The vertical dashed line is drawn at the CRE value for each individual agent. Cytotoxic response as represented by the fraction of YoYo positive cells in cOSA cell lines to (G) alisertib, (H) cabozantinib, (I) crizotinib, (J) dasatinib, (K) onvansertib and (L) sorafenib sorafenib following 96‐h continuous exposure to each drug. Open circles at each timepoint represent the average response of a given cell line and the green line represents the average spline curve for all cell lines combined. The vertical dashed line is drawn at the CRE value for each individual agent. (M) Heat map representing the relationship of the measured GR_50_ and the CRE for eight targeted agents and six hOSA cell lines. A lower GR_50_/CRE value represents greater sensitivity to a given agent relative to the CRE. (N) Heat map representing the fraction of dead cells at in vitro drug levels representing the CRE.

The activity of a subset of the targeted agents tested in the cOSA cell lines was tested against a panel of six hOSA cell lines. The agents tested were those that showed significant activity in the cOSA panel at the CRE (alisertib, crizotinib, onvansertib and sorafenib) as well as four other agents that have been tested clinically in human osteosarcoma patients that included cabozantinib [25], dasatinib [26] and palbociclib [27]. Defactinib was also included as a component of a currently ongoing clinical trial in canine metastatic OSA [28]. The GR response and cytotoxicity to these hOSA cell lines are shown in Figure 2A–2L and the GR_50_/CRE and fraction dead cells at the CRE represented as heatmaps in Figure 2M and Figure 2N. Like the response determined in the cOSA cell lines, the hOSA cell lines showed sensitivity to alisertib, crizotinib, onvansertib and sorafenib, and little response to other agents at the CRE.

### 3.4 Comparison of cOSA and hOSA Cell Lines

**FIGURE 3:**
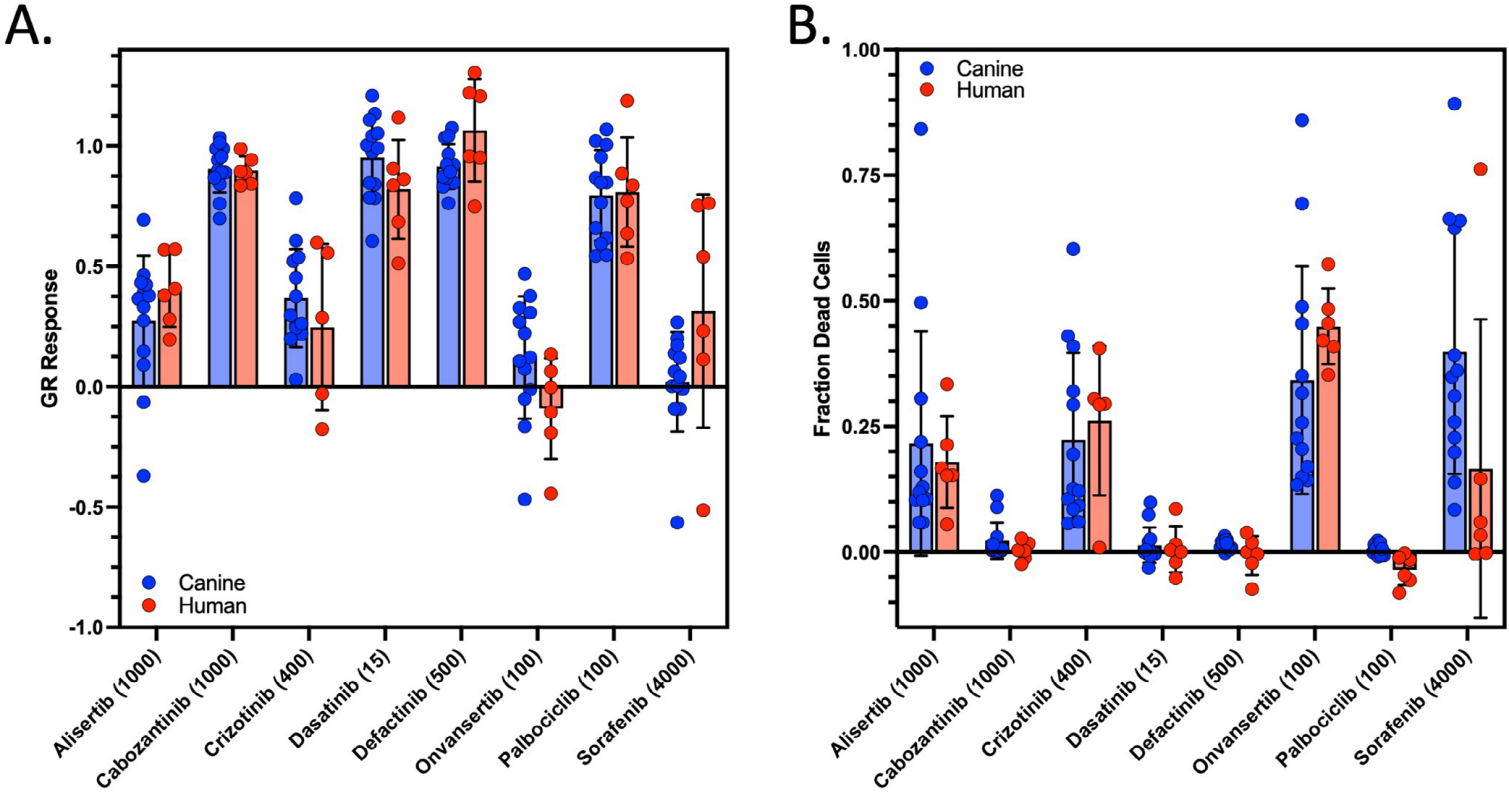
Comparative (A) GR response and (B) cytotoxicity of canine (blue) and human (red) osteosarcoma cell lines to eight targeted agents at in vitro CRE equivalent following 96‐h drug exposure. Circles represent the average response of individual cell lines and the bars represent the mean and standard deviation across the cell line panels. The number in parentheses next to the labels on the *x*‐axis is the in vitro CRE concentration in ng/mL for that drug.

The GR response and cytotoxicity for drugs screened in both the cOSA and hOSA cell lines at the CRE are shown in Figure 3. The range of responses in the 13 cOSA panel and the six hOSA cell lines are similar for each of the targeted agents. For example, the median GR value and range (minimum, maximum) across the cOSA panel for crizotinib at 400 ng/mL is 0.297 (0.030, 0.783) and in the hOSA cell lines is 0.287 (−0.176, 0.599), and for palbociclib at 100 ng/mL is 0.847 (0.543, 1.069) and 0.805 (0.533, 1.189) for the cOSA and hOSA cell lines, respectively. Comparison of the drug responses across the cOSA and hOSA panels showed no significant differences in GR response or cytotoxicity across the 8 drugs tested in both.

### 3.5 Correlations of Response in cOSA Cell Lines With Hallmark Pathways

Gene Set Variation analysis (GSVA) was conducted with RNASeq data for each of the cOSA cell lines in the screen and GSVA scores for each of the Hallmark gene sets calculated. Correlations of drug response (GR and cytotoxicity) at the CRE were determined for each of the drugs with significant activity versus the Hallmark pathways scores and heat maps representing the Hallmark scores ranked for correlation with GR response and cytotoxicity are shown in Figures S2 and S3 and pathways with significant correlations listed in Table 2.

**TABLE 2:** Significant hallmark pathway correlations with targeted agent response.

Targeted agent	Response	Hallmark pathway	Correlation *r* value	Correlation *p* value
Alisertib	GR GR GR GR Cyto	PROTEIN_SECRETIONHEME_METABOLISMFATTY_ACID_METABOLISMADIPOGENESIS—	0.5980 0.5653 0.5602 0.5535	0.0309 0.0441 0.0465 0.0497
Crizotinib	GR GR GR GR GR GR GR Cyto	APICAL_JUNCTION HEDGEHOG_SIGNALLING PI3K_AKT_MTOR_SIGNALLING E2F_TARGETS G2M_CHECKPOINT MYC_TARGETS_V1 MYC_TARGETS_V2 HEDGEHOG_SIGNALLING	0.5616 0.5604 0.5591 −0.6693 −0.6423 −0.6370 −0.5595 −0.5803	0.0458 0.0464 0.0470 0.0124 0.0179 0.0192 0.0468 0.0376
Onvansertib	GR GR GR Cyto Cyto	APICAL_JUNCTION IL2_STAT5_SIGNALLING MYC_TARGETS_V2 APICAL_JUNCTION IL2_STAT5_SIGNALLING	0.6730 0.5948 −0.5688 −0.6700 −0.6496	0.0117 0.032 0.0425 0.0122 0.0163
Sorafenib	GR Cyto	— —	— —	— —

### 3.6 Myc Score and Correlation to Crizotinib and Onvansertib Response in cOSA Cell Lines

**FIGURE 4:**
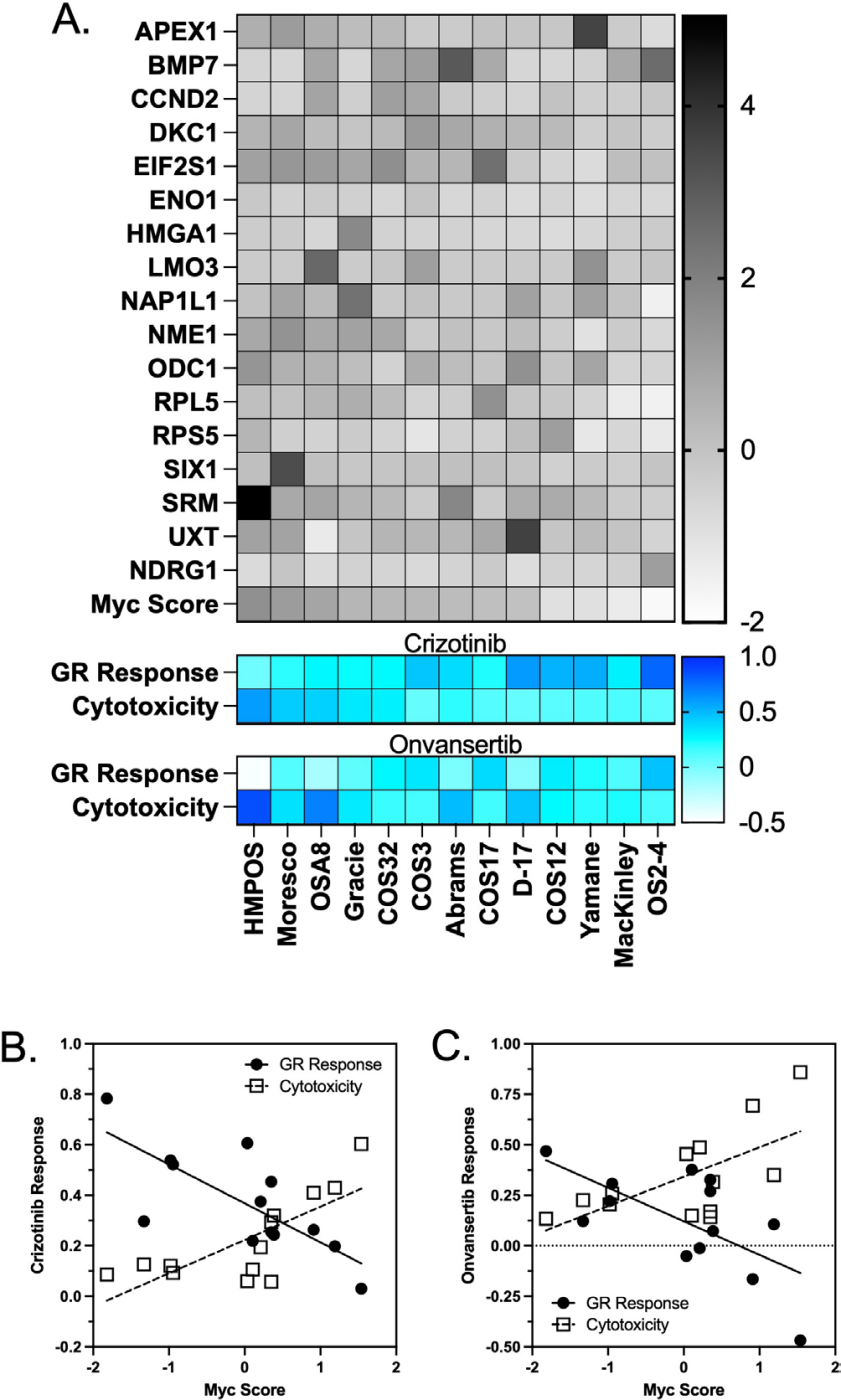
(A) Expression of genes used in the calculation of the Myc score in cOSA cell lines and GR response and cytotoxicity of cOSA cell lines to crizotinib and onvansertib. The cell lines are arranged from left to right based on calculated Myc score which is also represented on the heatmap. The correlation of GR response and cytotoxicity for crizotinib (B) and onvansertib (C) versus Myc score. The Spearman correlation for GR response (*r* = −0.7758, *p* = 0.0026) and cytotoxicity (*r* = −0.7758, *p* = 0.0026) for crizotinib is significant at *p* < 0.01. The Spearman correlation for GR response (*r* = −0.5722, *p* = 0.0439) and cytotoxicity (*r* = 0.5722, *p* = 0.0439) for onvansertib is significant at *p* < 0.05.

Correlation with Myc signalling for crizotinib and onvansertib was tested versus an independent Myc score based on expression of Myc target genes [20]. A heat map representing the relative expression of this gene set is shown in Figure 4A as well as the calculated Myc score and GR response and cytotoxicity to crizotinib and onvansertib at the CRE. The correlation of the Myc score to GR response and cytotoxicity for crizotinib and onvansertib is shown in Figure 4B,C where a higher MYC score leads to a decrease in growth rate (GR) and an increase in observed cytotoxicity. The Myc score significantly correlated to crizotinib GR response (*r* = −0.7758, *p* = 0.0026) and cytotoxicity (*r* = 0.7098, *p* = 0.0083) and to onvansertib GR response (*r* = −0.5722, *p* = 0.0439) and cytotoxicity (*r* = 0.5722, *p* = 0.0439).

### 3.7 Hedgehog Signalling and Response of cOSA Cell Lines to Crizotinib

**FIGURE 5:**
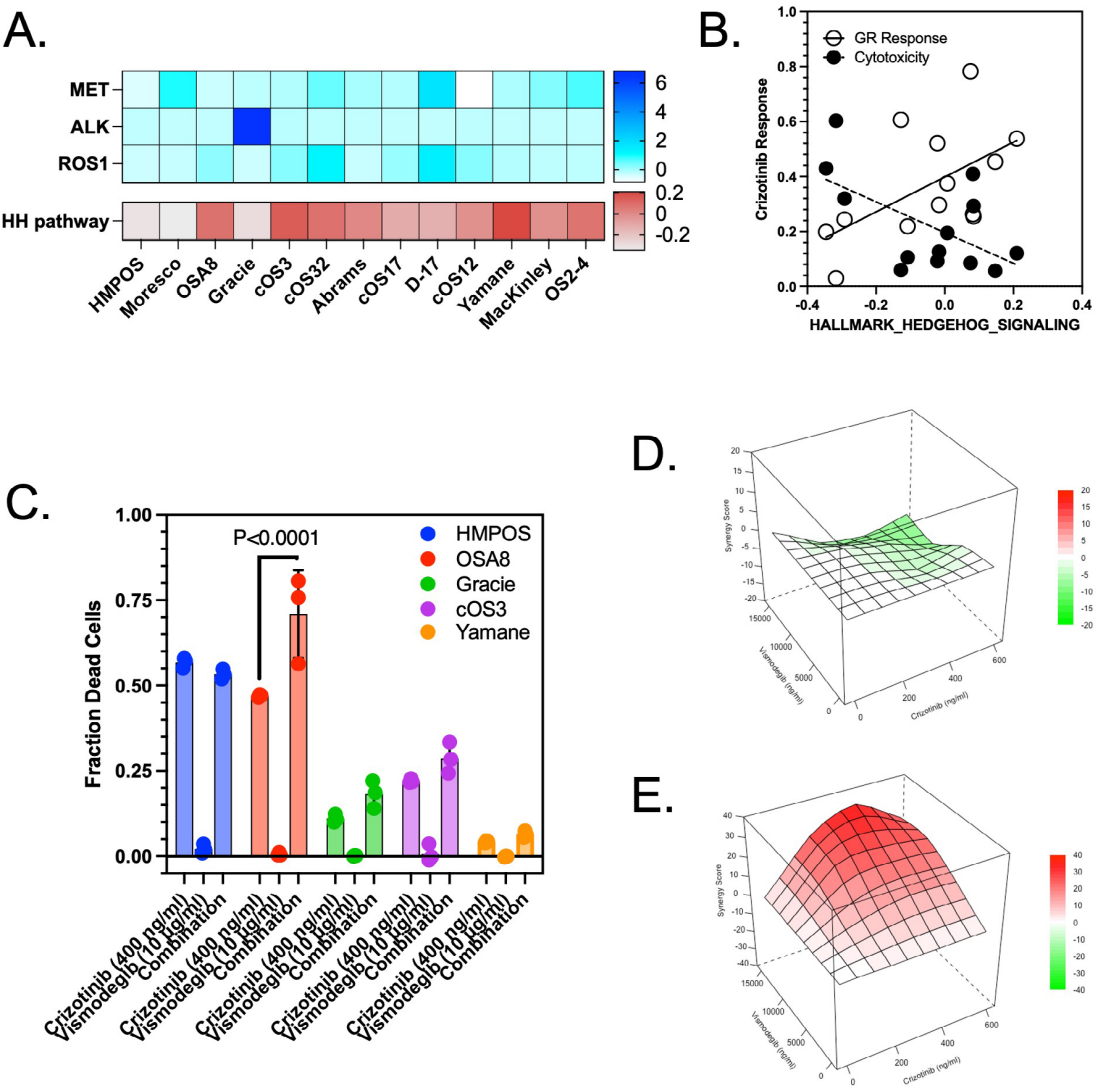
(A) Expression of crizotinib putative targets and HEDGEHOG_SIGNALLING hallmark pathway score in cOSA cell lines. Cell lines are arranged from left to right by increased sensitivity to crizotinib. (B) Correlation of HEDGEHOG_SIGNALLING hallmark score with GR response (*r* = 0.5604, *p* = 0.0464) and cytotoxicity (r = −0.5803, *p* = 0.0376) to crizotinib at the in vitro CRE. (C) Cytotoxicity of crizotinib (CRE = 400 ng/mL) and vismodegib (10 μg/mL) alone and in combination in HMPOS, OSA8, Gracie, cOS3 and Yamane cOSA cell lines at 96‐h of drug treatment. Bliss synergy analysis of combined crizotinib and vismodegib in (D) HMPOS and (E) OSA8 cOSA cell lines. Bliss analysis showed a mean synergy score of 20.2 in the OSA8 cell line (*p* = 4.05 × 10^−8^) and no significant synergy in the HMPOS cell line.

Both crizotinib GR response and cytotoxicity correlated with HEDGEHOG_SIGNALLING (Table 2 and Figure 5B). Shown in Figure 5A are the relative expression of putative target genes for crizotinib as well as the HEDGEHOG_SIGNALLING score arranged with respect to crizotinib sensitivity. The inclusion of the Hedgehog pathway inhibitor vismodegib at steady‐state concentrations (10 μg/mL) observed in dogs being treated with vismodegib (unpublished data) in an ongoing trial showed increased cytotoxicity induced by crizotinib in the OSA8 cell line that shows high Hedgehog signalling and significant response to crizotinib but did not increase the response in the HMPOS cell line with low Hedgehog signalling (Figure 5C). Vismodegib did not significantly increase the response to crizotinib in cell lines (Gracie, cOS3 and Yamane) that showed low cytotoxic response to crizotinib at the CRE regardless of HEDGEHOG_SIGNALLING pathway score. Analysis of synergy using Bliss Analysis showed significant synergy in the OSA8 cell line with a mean synergy score of 20.2 (*p* = 4.05 × 10^−9^) across the range of crizotinib and vismodegib concentrations tested while the HMPOS cell line showed no significant synergy (Figure 5D,E).

### 3.8 Onvansertib Response and Epithelial and Anti‐Apoptotic Score

**FIGURE 6:**
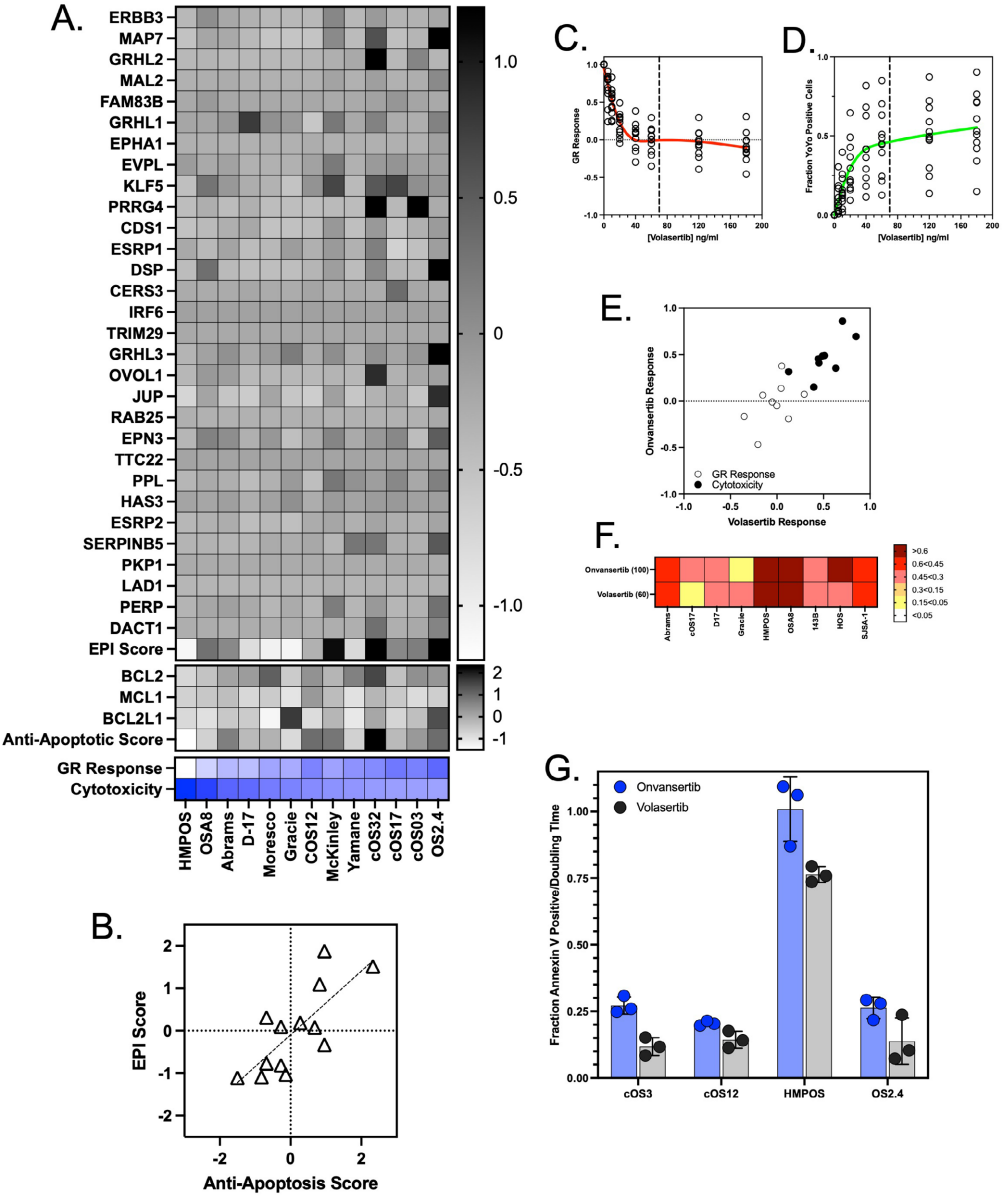
(A) Expression of EPI Score genes (top panel), anti‐apoptotic genes (middle panel) and onvansertib GR and cytotoxic response (bottom panel) at the in vitro CRE in cOSA cell lines. Cell lines are arranged left to right to decreased sensitivity to onvansertib and the EPI and Anti‐Apoptotic scores are included at the bottom of the respective gene expression panels. (B) Spearman correlation (*r* = 0.6953, *p* = 0.0083) of the EPI score with the Anti‐Apoptotic score in cOSA cell lines. GR (C) and cytotoxic (D) response of cOSA and hOSA cell lines to the polo like kinase 1 inhibitor volasertib following 96‐h exposure. Individual circles represent the mean value for each of the OSA cell lines and the red and green lines represent a spline of the mean response for GR and cytotoxic response, respectively. The vertical dashed line represents the estimated CRE for volasertib in paediatric patients dosed at the MTD of 200 mg/m^2^. (E) Correlation of onvansertib and volasertib response at the CRE for each agent (*r* = 0.8345, *p* < 0.0001) and the measured cytotoxic response (F) for each agent. (G) Annexin V positive cells per doubling time in response to onvansertib and volasertib at the in vitro CRE in cOS3, cOS12, HMPOS and OS2.4 cOSA cell lines. The HMPOS cell line had a significant increase in the Annexin V positive fraction versus the other cell lines (*p* < 0.001).

Although the EPITHELIAL_MESENCHYMAL_TRANSITION pathway was not significantly correlated with onvansertib response, the relationship between the apical junction, epithelial cell polarity and E‐cadherin signalling [29] led to an analysis of other epithelial and mesenchymal gene expression signatures due to APICAL_JUNCTION pathway score showing correlation with both onvansertib GR response and cytotoxicity (Table 2). The calculation of epithelial (EPI) and mesenchymal (MES) scores resulted in EPI scores that were highly correlative with the APICAL_JUNCTION scores of the cOSA cell lines (*r* = 0.7040, *p* = 0.0072). EPI score correlations with GR response (*r* = 0.5549, *p* = 0.0525) and cytotoxicity (−0.5934, *p* = 0.0360) at the CRE suggest some relationship with response; however, MES scores did not have any predictive values with GR response (*r* = 0.2568, *p* = 0.3970) and cytotoxicity (*r* = −0.2186, *p* = 0.4730). Relative expression of the genes used in the calculation of the EPI score are shown in Figure 6A. An analysis of three EMT transcription factors (Snail 1, Zeb1 and Twist 2) and the calculation of an EMT transcription score (Figure S4) show that the EMT transcription score has a negative relationship with the calculated EPI score (*r* = −0.4792, *p* = 0.0993) and calculated anti‐apoptotic score (−0.6408, *p* = 0.0183) and a positive relationship with onvansertib cytotoxicity (*r* = 0.7614, *p* = 0.0035). This adds further support to the relationship between EMT characteristics in the response of these cell lines to PLK1 inhibition.

### 3.9 Anti‐Apoptotic Score Correlations

Previous studies linking EMT to the response of NSCLC cell lines to PLK1 inhibitors showed that PLK1 inhibition led to a G_2_‐M arrest in all cell lines and that increased drug response was a function of increased apoptosis in sensitive cell lines [30]. Calculation of an anti‐apoptotic score based on the expression of a three‐gene panel of anti‐apoptotic proteins showed a strong correlation (*r* = 0.6953, *p* = 0.0083, Figure 6B) with the EPI score for the cOSA cell lines as well as correlating with onvansertib cytotoxicity at the CRE (*r* = −0.5868, *p* = 0.0350). Anti‐apoptotic score did not significantly correlate with the cytotoxic response of any of the other drugs that showed a significant response in the cOSA cell line panel. Expression of the genes used in the anti‐apoptotic score, the anti‐apoptotic score, and response of cOSA cell lines to onvansertib are shown in Figure 6A.

### 3.10 Response to Targeted Agents With the Same Putative Target

A subset of cOSA and hOSA cell lines were tested against another PLK1 inhibitor under clinical development, volasertib, to verify target response (Figures 6C and 6D). Shown in Figure 6E is the GR and cytotoxicity response at the CRE for onvansertib and volasertib (*r* = 0.8345, *p* < 0.0001), and in Figure 6F a heatmap comparing the cytotoxicity response at the CRE. Measurement of Annexin V positive cells was carried out in cells with low (HMPOS) and high (cOS3, cOS12, and OS2.4) anti‐apoptotic scores following treatment with onvansertib or volasertib at the calculated CRE (Figure 6G). The results show a significant increase in the fraction of Annexin V positive cells in the HMPOS cell line in response to either onvansertib or volasertib whereas the cell lines that are less sensitive to PLK1 inhibition showed fewer Annexin V positive cells.

## 4 Discussion

Treatment of osteosarcoma has remained unchanged for the last 30 years with platinum‐based chemotherapy, anthracyclines and methotrexate remaining the standard of care. The genomic heterogenous nature of the disease and the lack of druggable driving mutations has led to no clear druggable target coming to the forefront for OSA trials although targeted agents have been tested in paediatric OSA patients with mixed results. Our goal was to screen a set of 12 targeted agents against a panel of 13 cOSA cell lines to determine antiproliferative and cytotoxic activity while benchmarking the activity to drug concentrations that are established as clinically relevant exposures (CRE) in adult or paediatric patient populations. Drugs with identified activity at the CRE in at least a subset of the cOSA cell lines were then screened against a smaller set of hOSA cell lines to determine the cross‐species similarity in response.

The importance of pet dogs with OSA in the development of treatment strategies for the human disease is well established and has been thoroughly reviewed [1, 31]. The number of dogs with OSA is approximately 10‐fold that seen in humans, and the disease is pathologically and genetically very similar and follows the same pattern of progression. Therefore, canine OSA provides a population of surrogate patients for testing novel OSA treatment strategies with the added benefit that the animal population being treated may benefit as well from discovery of effective therapies.

The drugs with identified activity in our screen included alisertib, crizotinib, onvansertib, and sorafenib. The targets of alisertib and onvansertib, Aurora A Kinase (AurAK) and Polo‐Like Kinase 1 (PLK1), are kinases involved in cell division that are known to be overexpressed in cancers. Further, AurAK and PLK1 play reciprocal roles in the regulation of each other [32], but despite this interaction, there was no correlation in response to these agents. Previous studies have shown that response to AurAK inhibitors correlated to copy number variation in AurAK and Myc [33, 34]. An evaluation of RNASeq data does not see a correlation between response to alisertib and expression of either AurAK or Myc, suggesting that gene expression itself is not predictive. Further, neither of the MYC_TARGETS hallmarks gene sets nor the Myc score showed any correlation with alisertib response.

Crizotinib response showed an inverse correlation with MYC_TARGETS_V1 and MYC_TARGETS_V2 and this relationship held with the calculation of a score based on Myc target genes previously described to predict a poor prognosis in Myc‐driven tumours [20]. Other studies have shown that siRNA knockdown of MYC or inhibition using TMPyP4 can sensitize ALK positive NSCLC cells to crizotinib [35]. The inverse relationship observed in the cOSA cell lines suggests a difference in crizotinib response in non‐ALK driven tumour cells as 6/13 cOSA cell lines show no ALK transcript counts in RNASeq data. It should be noted that MYC scores showed no correlation with proliferation rates across the cOSA cell line panel (data not shown) and thus cell growth alone does not account for these differences in response. A decrease in crizotinib response correlated with HEDGEHOG_SIGNALLING scores. Previous studies have established that activation of the MET and hedgehog pathways can lead to resistance to EGFR targeted therapies [36, 37], however, this is the first report of hedgehog signalling potentially playing a role in determining response to a kinase inhibitor that includes MET as a target.

Response to onvansertib showed correlations to Myc signalling as well as to genes regulated by E‐cadherin (EPI score). Higher Myc signalling correlated to decreased growth (lower GR) and increased cytotoxicity. For the APICAL_JUNCTION and EPI score, higher scores correlated to decreased response which is consistent with responses in non‐small cell lung cancer (NSCLC) cell lines where high epithelial‐mesenchymal (EMT) scores were predictive of response to PLK1 inhibition and E‐cadherin expression was significantly higher in the resistant cell lines [30]. Sensitivity of NSCLC cell lines in this study was dependent on increased apoptosis in the higher EMT group. The relationships between EPI score, anti‐apoptotic score, response to PLK1 inhibitors and increased apoptosis in cOSA cell lines suggest a similar relationship in OSA.

Response to sorafenib in liver cancer patients has been correlated to KDR, PDGFRB, KIT, RAF1, EGFR, MTOR and FGFR1 gene expression in the tumours [38]. Correlation of GR or cytotoxic response with the expression of these genes across the cOSA panel showed no significant correlations. Although there were no significant correlations with the expression of these genes, the sorafenib target genes (KDR, PDGFRB, KIT, RAF) all showed r values consistent with increased expression leading to an increase in response whereas non‐target genes that have been identified as potentially leading to resistance [39, 40, 41, 42, 43] (EGFR, MTOR, FGFR1) had r values showing inverse relationships. Preliminary studies in cOSA and hOSA cell lines with combinations of sorafenib with EGFR (sapitinib), mTOR (Torin1) and FGFR (erdafitinib) inhibitors show enhanced sorafenib response to these combinations in select cases (data not shown) suggesting that complex interactions mediate the sensitivity and resistance to this multi‐kinase inhibitor.

Kinase inhibitors have been used in human osteosarcoma patients and in general, the multi‐target kinase inhibitors including apatinib [44], regorafenib [45], anlotinib [46], cabozantanib [25], and sorafenib [47] are the most active although some responses have also been observed with the VEGF receptor targeting drugs cedarinib [48], and axitinib [49]. None of these trials included patient stratification based on any known biomarkers which has been suggested to be a critical component to understanding response heterogeneity and the magnitude of therapeutic benefit associated with independent drug action [50]. The studies presented here identify potential genomic indicators of tumour cell response in a canine panel of OSA cell lines at drug concentrations achievable in humans. The limitations of these studies are that the genomic markers identified in cell lines have not been validated in tumours with the added complexity of the tumour microenvironment and are solely based on transcript levels. An obvious next step is assessment of these pathway differences in canine OSA tumours and identification of appropriate biomarkers for testing in a canine patient population. Safety testing in Phase 0 or Phase 1 trials of the agents identified as having activity would include pharmacokinetic studies for comparison to the CRE estimated from the human data. A case study of dasatinib in a dog with OSA showed steady‐state plasma levels very similar to those observed in human patients [51], and lapatinib steady‐state exposure in dogs is estimated to be approximately equal to human exposure at recommended safe doses in each species [52, 53]. These results suggest that the CRE exposures in humans approximate clinical dosing exposure in dogs for at least a subset of targeted agents.

In conclusion, we have carried out a screen of 12 targeted agents against a panel of 13 canine and 6 human osteosarcoma cell lines and identified drugs and drug classes with significant in vitro activity at concentrations relevant to levels measured in patients at clinically used doses and dosing regimens. Additionally, correlations with genomics‐based pathways have identified potential biomarkers of response for further study.

## Author Contributions

Conceptualization: Daniel L. Gustafson. Data Curation: Daniel L. Gustafson, Kala A. Early, Maritza A. Morales, Klaudia A. Poplawski, Sunetra Das, Dawn L. Duval. Resources: Daniel L. Gustafson, Dawn L. Duval. Writing – Original Draft: Daniel L. Gustafson, Writing – Reviewing and Editing: Daniel L. Gustafson. Supervision: Daniel L. Gustafson, Dawn L. Duval.

## Funding

This work was supported by the Shipley University Chair in Comparative Oncology awarded to DL Gustafson, the Flint Animal Cancer Center, the Anschutz Foundation, and a TUNE fellowship awarded to K Poplawski. RNA sequencing was carried out by the Genomics Shared Resource of the University of Colorado Cancer Center (P30 CA046934).

## Ethics Statement

The authors have nothing to report.

## Conflicts of Interest

The authors declare no conflicts of interest.

## Supporting information


**Data S1:** Calculation of Clinically Relevant Exposures (CRE).

**Figure S1:** Estimated steady‐state plasma levels of (A) crizotinib and (B) sorafenib dosed at 280 and 200 mg/m^2^, respectively, every 12 h. The clinically relevant exposure (CRE) used for in vitro studies is shown as a horizontal dashed line for each curve and represents the estimated average plasma concentration at steady‐state (C_ss,avg_) across a dosing interval as estimated from reported clearance values for crizotinib and sorafenib in paediatric populations. The equation used for calculation of the CRE is shown in section 2.3. in the Materials and Methods.

**Figure S2:** Correlation heatmaps of hallmark pathways with GR response at the CRE for (A) alisertib, (B) crizotinib, (C) onvansertib and (D) sorafenib. Cell lines are arranged from left to right with decreasing sensitivity for each of the individual drugs. Pathways with significant correlations (*p* < 0.05) with response are listed in Table 2. Correlation of drug response with hallmark pathways scores were done using Pearson correlation as described in the Materials and Methods.

**Figure S3:** Correlation heatmaps of hallmark pathways with cytotoxic response at the CRE for (A) alisertib, (B) crizotinib, (C) onvansertib and (D) sorafenib. Cell lines are arranged from left to right with increasing sensitivity for each of the individual drugs. Pathways with significant correlations (*p* < 0.05) with response are listed in Table 2. Correlation of drug response with hallmark pathways scores were done using Pearson correlation as described in the Materials and Methods.

**Figure S4:** (A) Relative gene expression of EMT Transcription score genes and EMT Transcription score with onvansertib sytotoxicity in cOSA cell lines. The cell lines are arranged left to right with increasing sensitivity to onvansertib. Correlation of EMT Transcription score with (B) EPI score, (C) Anti‐Apoptotic score and (D) Onvansertib cytotoxicity in cOSA cell lines.

**Table S1:** Molecular Characteristics of Canine Osteosarcoma Cell Lines.

**Table S2:** Molecular Characteristics of Human Osteosarcoma Cell Lines.

## Data Availability

The data that are presented in this manuscript and support the findings of the study are available from the corresponding author upon reasonable request.
