# Supplementary material for: Comparative Response of Canine and Human Osteosarcoma Tumour Cell Lines to Molecularly Targeted Anticancer Agents at Clinically Relevant Exposures With Analysis of Genomic Biomarkers
Source: Vet Comp Oncol. 2026 Apr 26;24(3):532–44. doi: 10.1111/vco.70074 (PMC13456611; doi:10.1111/vco.70074)
Supplement: Supplementary file 1 [file VCO-24-532-s002.docx]

**Calculation of Clinically Relevant Exposures (CRE)**

**Drugs Dosed Daily or More Frequently**

**Aliseritib.** The pharmacokinetics of alisertib were reviewed in both adult [1, 2] and pediatric patients [3]. Using a dose of 50 mg BID in adult patients and 80 mg/m^2^ daily in pediatric patients, the average steady-state plasma concentration (C_ss,avg_) over a dosing interval was estimated to be 779 ng/ml in adult patients and 1510 ng/ml in pediatric patients. Using these values the estimated CRE is 1150 ng/ml.

**Alpelisib.** The pharmacokinetics of alpelisib dosed orally at 30-450 mg daily were used for a population pharmacokinetic model [4]. A population based estimated clearance of 11.5 L/h was used to calculate the C_ss,avg_ at daily doses of 50 mg and 250 mg which were estimated at 181 and 906 ng/ml, respectively. Using these values the estimated CRE is approximately 500 ng/ml.

**Cabozantinib.** The pharmacokinetics of cabozantinib have been reported in both adult [5] and pediatric populations [6]. The use of repeated dose data at 140 and 100 mg per day lead to an estimated clearance rate of 3.55 L/h in adults and doses of 30-55 mg/m^2^/day in pediatric patients gave an estimated clearance of 1.68 L/h/m^2^. Using the recommended doses of 60 mg for adults and 40 mg for pediatric patients (BSA<1.2 m^2^) daily, the estimated C_ss,avg_ is 704 ng/ml for adults and 825 ng/ml for pediatric patients (BSA=1.0) and an estimated CRE of 765 ng/ml.

**Crizotinib.** The pharmacokinetics of crizotinib has been determined in several pediatric patient populations [7-10] with clearance rates reported from 43.8 to 79.2 L/h/m^2^. Using a recommended dose of 280 mg/m^2^ BID, the C_ss,avg_ and CRE across the included studies is calculated to be 415 ng/ml.

**Dasatinib.** The pharmacokinetics of dasatinib has been determined in both adult [11] and pediatric patients [12]. The estimated clearance in adult and pediatric patients for dasatinib was 249 L/h and 246 L/h/m^2^, respectively. Using an adult dose of 100 mg and a pediatric dose estimated at 70 mg/m^2^ , the C_ss,avg_ values are calculated as 16.7 and 11.9 ng/ml to give an estimated CRE of 14.3 ng/ml.

**Defactinib.** The pharmacokinetics of defactinib has been reported in an adult Japanese population [13] and a predominantly Caucasian cohort [14]. The estimated clearance at steady-state in these patient groups were 75 L/h and 119 L/h leading to C_ss,avg_ of 280 and 444 ng/ml and an estimated CRE of 362 ng/ml.

**Onvansertib.** The pharmacokinetics of onvansertib has been reported in adult populations in combination with other therapies [15, 16] and the estimated steady-state clearances calculated as 11.0 and 7.9 L/h/m^2^. With a recommended Phase II dose of 24 mg/m^2^, the calculated C_ss,avg_ are 91 and 127 ng/ml and an estimated CRE of 109 ng/ml.

**Palbociclib.** The pharmacokinetics of palbociclib in pediatric patients has been reported in multiple studies [17-19] with reported steady-state clearances of 36.8, 40.4 and 28.5 L/h/m2 at doses ranging from 50 to 95 mg/m^2^. At a dose of 75 mg/m^2^/day, the C_ss,avg_ values are estimated at 85, 77 and 110 ng/ml and an estimated CRE of 91 ng/ml.

**Sapitinib.** The pharmacokinetics of sapitinib has been reported in Japanese [20] and Caucasian [21] adult populations with reported steady-state clearance values of 31 and 52 L/h, respectively. With a dose of 160 mg BID, this leads to estimated C_ss,avg_ values of 427 and 256 ng/ml and a CRE value of 342 ng/ml.

**Sorafenib.** The pharmacokinetics of sorafenib have been evaluated in pediatric populations [22] and steady-state clearance reported as 3.5, 3.8 and 4.4 L/h/m^2^ in separate cohorts. Calculated C_ss,avg_ at a dose of 200 mg/m^2^ BID are 4817, 4398 and 3823 ng/ml and an estimated CRE of 4307 ng/ml.

**Vactosertib.** The pharmacokinetics of vactosertib have been evaluated in adult patient populations at dose ranges from 30-340 mg daily and analyzed using both non-compartmental [23] and population-based approaches [24]. The reported clearance values ranged from 29-32 L/h and no accumulation was reported after multiple doses, consistent with the reported short terminal half-life of approximately 3 h. The use of a clearance value of 30 L/h and a recommended phase II dose of 300 mg BID [25] gives an estimated C_ss,avg_ and CRE value of 833 ng/ml.

**Venetoclax.** The pharmacokinetics of venetoclax have been well described in adult populations [26, 27] with multiple population-based analyses [28-30] reporting clearance values ranging from 15.0 to 19.5 L/h. Venetoclax pharmacokinetics have also been studied in pediatric populations with a recommendation for dosing in these patients to target drug exposures of 400-600 mg daily dosing in adults [31]. Using an estimated clearance value of 17.3 L/h and doses of 400 and 600 mg daily, C_ss,avg_ values are estimated at 963 and 1,445 ng/ml, respectively, and a CRE of 1,204 ng/ml.

**Drugs Dosed Weekly or Less Frequently**

**Volasertib.** Volasertib pharmacokinetics have been studied in both adult [32] and pediatric populations [33]. Drug dosing was done every 21 days in the adult population with an MTD of 300 mg per week, and in pediatric patients an MTD of 200 mg/m^2^ was established when dosed every 14 days. The lack of daily dosing with this drug makes estimation of a CRE in tissue culture more difficult to assess. However, drug concentrations in these patient populations at 24, 48 and 96 hours post-dosing were reported and were 50-75 ng/ml, 25-30 ng/ml and 10-20 ng/ml, respectively. Reported AUC values at the MTD doses for both patient populations were in the range of 6000-7000 ng/ml x hr. Thus, for a 96 hour drug exposure in tissue culture of 70 ng/ml total drug exposure would approximate the exposure for a single dose in patients.

1.	Cervantes, A., et al., *Phase I pharmacokinetic/pharmacodynamic study of MLN8237, an investigational, oral, selective aurora a kinase inhibitor, in patients with advanced solid tumors.* Clin Cancer Res, 2012. **18**(17): p. 4764-74.

2.	Zhou, X., et al., *Pharmacokinetics of the Investigational Aurora A Kinase Inhibitor Alisertib in Adult Patients With Advanced Solid Tumors or Relapsed/Refractory Lymphoma With Varying Degrees of Hepatic Dysfunction.* J Clin Pharmacol, 2019. **59**(9): p. 1204-1215.

3.	Zhou, X., et al., *Population Pharmacokinetics and Exposure-Safety Relationships of Alisertib in Children and Adolescents With Advanced Malignancies.* J Clin Pharmacol, 2022. **62**(2): p. 206-219.

4.	De Buck, S.S., et al., *Population pharmacokinetics and pharmacodynamics of BYL719, a phosphoinositide 3-kinase antagonist, in adult patients with advanced solid malignancies.* Br J Clin Pharmacol, 2014. **78**(3): p. 543-55.

5.	Lacy, S.A., D.R. Miles, and L.T. Nguyen, *Clinical Pharmacokinetics and Pharmacodynamics of Cabozantinib.* Clin Pharmacokinet, 2017. **56**(5): p. 477-491.

6.	Chuk, M.K., et al., *A phase 1 study of cabozantinib in children and adolescents with recurrent or refractory solid tumors, including CNS tumors: Trial ADVL1211, a report from the Children's Oncology Group.* Pediatric Blood & Cancer, 2018. **65**(8): p. e27077.

7.	Jerry, L., et al., *Population modeling analyses of crizotinib in pediatric patients with ALK-positive advanced cancers.* Pediatr Blood Cancer, 2024. **71**(9): p. e31139.

8.	Gibson, E.G., et al., *Population pharmacokinetic analysis of crizotinib in children with progressive/recurrent high-grade and diffuse intrinsic pontine gliomas.* Cancer Chemother Pharmacol, 2021. **88**(6): p. 1009-1020.

9.	Greengard, E., et al., *Safety, tolerability and pharmacokinetics of crizotinib in combination with cytotoxic chemotherapy for pediatric patients with refractory solid tumors or anaplastic large cell lymphoma (ALCL): a Children's Oncology Group phase 1 consortium study (ADVL1212).* Cancer Chemother Pharmacol, 2020. **86**(6): p. 829-840.

10.	Balis, F.M., et al., *First-dose and steady-state pharmacokinetics of orally administered crizotinib in children with solid tumors: a report on ADVL0912 from the Children's Oncology Group Phase 1/Pilot Consortium.* Cancer Chemother Pharmacol, 2017. **79**(1): p. 181-187.

11.	Haura, E.B., et al., *Phase I/II study of the Src inhibitor dasatinib in combination with erlotinib in advanced non-small-cell lung cancer.* J Clin Oncol, 2010. **28**(8): p. 1387-94.

12.	Aplenc, R., et al., *Pediatric phase I trial and pharmacokinetic study of dasatinib: a report from the children's oncology group phase I consortium.* J Clin Oncol, 2011. **29**(7): p. 839-44.

13.	Shimizu, T., et al., *A first-in-Asian phase 1 study to evaluate safety, pharmacokinetics and clinical activity of VS-6063, a focal adhesion kinase (FAK) inhibitor in Japanese patients with advanced solid tumors.* Cancer Chemother Pharmacol, 2016. **77**(5): p. 997-1003.

14.	Jones, S.F., et al., *A phase I study of VS-6063, a second-generation focal adhesion kinase inhibitor, in patients with advanced solid tumors.* Invest New Drugs, 2015. **33**(5): p. 1100-7.

15.	Zeidan, A.M., et al., *A Phase Ib Study of Onvansertib, a Novel Oral PLK1 Inhibitor, in Combination Therapy for Patients with Relapsed or Refractory Acute Myeloid Leukemia.* Clin Cancer Res, 2020. **26**(23): p. 6132-6140.

16.	Ahn, D.H., et al., *Onvansertib in Combination with FOLFIRI and Bevacizumab in Second-Line Treatment of KRAS-Mutant Metastatic Colorectal Cancer: A Phase Ib Clinical Study.* Clin Cancer Res, 2024. **30**(10): p. 2039-2047.

17.	Panetta, J.C., et al., *Population Pharmacokinetic and Pharmacodynamic Study of Palbociclib in Children and Young Adults with Recurrent, Progressive, or Refractory Brain Tumors.* Pharmaceutics, 2024. **16**(12).

18.	Raetz, E.A., et al., *Palbociclib in combination with chemotherapy in pediatric and young adult patients with relapsed/refractory acute lymphoblastic leukemia and lymphoma: A Children's Oncology Group study (AINV18P1).* Pediatr Blood Cancer, 2023. **70**(11): p. e30609.

19.	Van Mater, D., et al., *A phase I trial of the CDK 4/6 inhibitor palbociclib in pediatric patients with progressive brain tumors: A Pediatric Brain Tumor Consortium study (PBTC-042).* Pediatr Blood Cancer, 2021. **68**(4): p. e28879.

20.	Kurata, T., et al., *Inhibition of EGFR, HER2 and HER3 signaling with AZD8931 alone and in combination with paclitaxel: phase i study in Japanese patients with advanced solid malignancies and advanced breast cancer.* Invest New Drugs, 2014. **32**(5): p. 946-54.

21.	Tjulandin, S., et al., *Phase I, dose-finding study of AZD8931, an inhibitor of EGFR (erbB1), HER2 (erbB2) and HER3 (erbB3) signaling, in patients with advanced solid tumors.* Invest New Drugs, 2014. **32**(1): p. 145-53.

22.	Widemann, B.C., et al., *A phase I trial and pharmacokinetic study of sorafenib in children with refractory solid tumors or leukemias: a Children's Oncology Group Phase I Consortium report.* Clin Cancer Res, 2012. **18**(21): p. 6011-22.

23.	Jung, S.Y., et al., *Pharmacokinetic characteristics of vactosertib, a new activin receptor-like kinase 5 inhibitor, in patients with advanced solid tumors in a first-in-human phase 1 study.* Invest New Drugs, 2020. **38**(3): p. 812-820.

24.	Jung, S.Y., et al., *Population pharmacokinetics of vactosertib, a new TGF-beta receptor type Iota inhibitor, in patients with advanced solid tumors.* Cancer Chemother Pharmacol, 2020. **85**(1): p. 173-183.

25.	Jung, M., et al., *1453P Safety and efficacy of vactosertib, a TGF-βR1 kinase inhibitor, in combination with paclitaxel in patients with metastatic gastric adenocarcinoma.* Annals of Oncology, 2020. **31**: p. S912.

26.	Roberts, A.W., et al., *Targeting BCL2 with Venetoclax in Relapsed Chronic Lymphocytic Leukemia.* N Engl J Med, 2016. **374**(4): p. 311-22.

27.	Minocha, M., et al., *Pharmacokinetics of the B-Cell Lymphoma 2 (Bcl-2) Inhibitor Venetoclax in Female Subjects with Systemic Lupus Erythematosus.* Clin Pharmacokinet, 2018. **57**(9): p. 1185-1198.

28.	Jones, A.K., et al., *Clinical Predictors of Venetoclax Pharmacokinetics in Chronic Lymphocytic Leukemia and Non-Hodgkin's Lymphoma Patients: a Pooled Population Pharmacokinetic Analysis.* AAPS J, 2016. **18**(5): p. 1192-1202.

29.	Deng, R., et al., *Bayesian Population Model of the Pharmacokinetics of Venetoclax in Combination with Rituximab in Patients with Relapsed/Refractory Chronic Lymphocytic Leukemia: Results from the Phase III MURANO Study.* Clin Pharmacokinet, 2019. **58**(12): p. 1621-1634.

30.	Gong, J.Q.X., et al., *Pooled Population Pharmacokinetic Analyses of Venetoclax in Patients Across Indications and Healthy Subjects from Phase 1, 2, and 3 Clinical Trials.* J Clin Pharmacol, 2023. **63**(8): p. 950-960.

31.	Badawi, M., et al., *Dosing of Venetoclax in Pediatric Patients with Relapsed Acute Myeloid Leukemia: Analysis of Developmental Pharmacokinetics and Exposure-Response Relationships.* Clinical Therapeutics, 2024. **46**(10): p. 759-767.

32.	Lin, C.C., et al., *A phase I study of two dosing schedules of volasertib (BI 6727), an intravenous polo-like kinase inhibitor, in patients with advanced solid malignancies.* Br J Cancer, 2014. **110**(10): p. 2434-40.

33.	Doz, F., et al., *Phase I dose-escalation study of volasertib in pediatric patients with acute leukemia or advanced solid tumors.* Pediatr Blood Cancer, 2019. **66**(10): p. e27900.
