# Supplementary material for: Comparative Response of Canine and Human Osteosarcoma Tumour Cell Lines to Molecularly Targeted Anticancer Agents at Clinically Relevant Exposures With Analysis of Genomic Biomarkers
Source: Vet Comp Oncol. 2026 Apr 26;24(3):532–44. doi: 10.1111/vco.70074 (PMC13456611; doi:10.1111/vco.70074)
Supplement: Supplementary file 2 [file VCO-24-532-s003.docx]

Supplementary Table S1. Molecular Characteristics of Canine Osteosarcoma Cell Lines

| **Cell Line Name** | **^a^Whole Exome Sequence/ Putative Driver Mutation** | **^b^Transcript Markers**  **RNA-Seq** |
| --- | --- | --- |
| Abrams | TP53 R301W, C329F | PTEN* Loss |
| D-17 | KRAS E157K | NOTCH1 (2), CDKN2A* Loss, MDM2 gain, SUFU--GBF1 fusion, VIM--NPM1 fusion |
| Gracie | SRGAP3, TP53 R300Q | CDKN2A Loss (24 counts) |
| HMPOS | TP53 R226H | PTEN* Loss |
| McKinley | MED12 L1938Cfs*48, PPM1D E472* | NOTCH1 (31), CDKN2A* Loss, |
| Moresco | NRAS Q61H, RAC1 P184T , TP53 R300Q | CDKN2A Loss* |
| OS2.4 | TP53 I284T, DAXX R229Efs*3 | RB1 Loss (178 counts), RB1--MLNR fusion |
| OSA8 | TP53 R300Q, KDM6A T1020Rfs*2, INPPL1 S590* | RB1 (163 counts), PTEN* Loss |
| FACC-18COS03 | TP53 P77L fs*2 and K171R, SMAD3 A112Rfs*87 | CDKN2A* Loss |
| FACC-19COS12 |  | CDKN2A* Loss, MDM4 gain, REV3L--SCG5 fusion |
| FACC-19COS17 | PRKCI,p.Arg587Cys ; TP53,Q370Afs*19 | CDKN2A* Loss |
| FACC-19COS32 | TP53 K171R, SMAD3 A112Rfs*87 | CDKN2A* Loss |
| Yamane | TP53 R232C, BRCA2 E3000K | CDKN2A* Loss |
| a- Whole exome sequencing and putative driver mutation is from Das et al.^16^ b- For transcript markers, genes were listed if the z-score was >±1.96 except for CDKN2A where cut off was -1.2. Raw counts of zero are indicated with * | | |
