# Supplementary material for: Comparative Response of Canine and Human Osteosarcoma Tumour Cell Lines to Molecularly Targeted Anticancer Agents at Clinically Relevant Exposures With Analysis of Genomic Biomarkers
Source: Vet Comp Oncol. 2026 Apr 26;24(3):532–44. doi: 10.1111/vco.70074 (PMC13456611; doi:10.1111/vco.70074)
Supplement: Supplementary file 3 [file VCO-24-532-s004.docx]

Supplementary Table S2. Molecular Characteristics of Human Osteosarcoma Cell Lines

| **Cell Line Name** | **Whole Exome/Genome Sequence/ Putative Driver mutation /CNV** | **Transcript Markers**  **RNA-Seq** |
| --- | --- | --- |
| 143B | KRAS A59T, G12S; TP53 R156P; Loss CDKN2A/B, RAD51D, NF1, MTAP, HOXA11, CDC73, SUZ12, EPHA3, SLFN11, FHIT, JARID2, BCOR, KDM5D; Gain MCL1, AURKA, MET, ZNF217, TPM3, JAK2, FGF1, ATP1A1, AR, TAF1 |  |
| HOS | TP53 R156P, MED12 X368_splice; Loss MTAP, NF1, RAD51D, CDKN2A/B, HOXA11, CDC73, KMT2C, SUZ12, EZH2,EPHA3, SLFN11, SMAD4, FHIT, SMAD2, PMAIP1, KDM5D, KEAP1; Gain MET, KAT7, MCL1, ZNF217, FGF19, RICTOR, TERT, CCND1, TPM3, FGF4, FGF3, YAP1, FGF1, CRLF2 | EXOC2-MET fusion, ERG fusion |
| MG63 | PTPN13 Q1647Tfs*13; Loss CDKN2A/B, MTAP, KDM5D, DUSP22, PTPRD, HLA-A; Gain PDGFRA, TERT, BRD4, AGO2, RNASEH2A, MEF2B, PAX5, MYC, ECSIT | TP53-ADGRE1 Fusion, TP53-VAV1 Fusion, MTAP fusions, JARID2 fusion |
| SaOS2 | RB1 X605_splice, ARID4B T1001Nfs*6, CAMTA X101_splice, ANKRD11 L187Rfs*17 | SAT2—TP53, SHBG—TP53, RB1 fusions |
| SJSA-1 | TP53 R273H; NRAS Q61K; KRAS Q61H, ATRX L359Tfs*3; KMT2D Y2199Ifs*65; Loss MTAP, CDKN2A/B; ATM, MRE11, CHEK1, PTPRD, SMARCA2, FANCG, SLIT3, CBL, PHOX2B, INPPL1, EED, BIRC3, SDHD, MED12, KMT2A, KDM5D; Gain MDM2, CDK4, TERT, CCND1/2, FGF3, IRS2, FGF19, HMGA2, FGF4, GLI1, PPM1D, KAT7, DDX5, COL1A1 | ENO1—ACTG1 fusion |
| U2OS | PPM1D R458*, BACH2 N72Kfs*2, Loss ATRX, CDC73, DUSP22, KMT2C, LRP1B, EZH2, KDM5D; Gain CCNE1, PPM1D, RAC1, AGO2, CDK6, ERBB3, MYC (no increase in expression), CARD11, EPHB4, MN1, TYMS, YES1, POU3F4, AR, TAF1 | PTPN2—PSMG2 fusion, RAD50—ZNF536 fusion |

- CNV are colored with corresponding change in transcript if available. Blue Loss, Red Gain
